# Depression screening and education: an examination of mental health literacy and stigma in a sample of Hispanic women

**DOI:** 10.1186/s12889-018-5516-4

**Published:** 2018-05-22

**Authors:** Veronica Lopez, Katherine Sanchez, Michael O. Killian, Brittany H. Eghaneyan

**Affiliations:** 1 School of Social Work, University of Texas at Arlington, 211 South Cooper Street, Arlington, TX 76019 USA; 2 Center for Applied Health Research, Baylor Scott and White Research Institute, 8080 North Central Expressway, Suite 1050, Dallas, TX 75206 USA

**Keywords:** Depression, Education, Hispanics, Stigma, Primary care, Mental health literacy

## Abstract

**Background:**

Mental health literacy consists of knowledge of a mental disorder and of the associated stigma. Barriers to depression treatment among Hispanic populations include persistent stigma which is primarily perpetuated by inadequate disease literacy and cultural factors. U.S.-born Hispanics are more likely to have depression compared to Hispanics born in Latin America and are less likely to follow a treatment plan compared to non-Hispanic whites. Hispanic women are more likely to access treatment through a primary care provider, making it an ideal setting for early mental health interventions.

**Methods:**

Baseline data from 319 female Hispanic patients enrolled in Project DESEO: Depression Screening and Education: Options to Reduce Barriers to Treatment, were examined. The study implemented universal screening with a self-report depression screening tool (the 9-item Patient Health Questionnaire (PHQ-9) and took place at one federally qualified health center (FQHC) over a 24-month period. The current analysis examined the relationship between four culturally adapted stigma measures and depression knowledge, and tested whether mental health literacy was comparable across education levels in a sample of Hispanic women diagnosed with depression.

**Results:**

Almost two-thirds of the sample had less than a high school education. Depression knowledge scores were significantly, weakly correlated with each the Stigma Concerns About Mental Health Care (*ρ* = − .165, *p* = .003), Latino Scale for Antidepressant Stigma (*p* = .124, *p* = .028), and Social Distance scores (*p* = .150, *p* = .007). Depression knowledge (F[2, 312] = 11.82, *p* < .001, partial *η*^2^ = .071), Social Distance scores (F[2, 312] = 3.34, *p* = .037, partial *η*^2^ = .021), and antidepressant medication stigma scores (F[2, 312] = 3.33, *p* = .037, partial *η*^2^ = .015) significantly varied by education category. Participants with at least some college education reported significantly greater depression knowledge and less stigma surrounding depression and medication than participants with lower education levels.

**Conclusions:**

Primary care settings are often the gateway to identifying undiagnosed mental health disorders, particularly for Hispanic women with comorbid physical health conditions. This study is unique in that it aims to examine the specific role of patient education level as a predictor of mental health literacy. For Hispanic women, understanding the mental health literacy of patients in a healthcare setting may improve quality of care through early detection of symptoms, culturally effective education and subsequent engagement in treatment.

**Trial registration:**

The study was registered with https://clinicaltrials.gov/: NCT02491034 July 2, 2015.

## Background

### Health and mental health literacy

Health literacy is a term used in public health to explain an individual’s capacity to understand health related information [1]. In healthcare settings, it is useful for professionals to understand patients’ health literacy level to deliver more effective health education that might prevent negative health outcomes [2]. Mental health literacy is similar to health literacy but refers specifically to mental health conditions and the belief systems that create stigma surrounding engagement in mental health treatment [3]. Mental health literacy consists of knowledge of a mental disorder, knowledge of stigma associated with mental illness, and the patient’s willingness to seek treatment [4].

Jorm [5] introduced a conceptual framework for understanding mental health literacy, with a focus on the stigma associated with mental illness which may influence engagement and adherence to treatment. According to Jorm’s framework, the three components of mental health literacy include: (1) *recognition* (of disorders and types of distress); (2) *knowledge* (of mental illness, self-help, professional help, and where to find information); (3) *attitudes/beliefs* (that promote self-help and may influence treatment outcomes) [5].

Quantitative measurement of mental health literacy in primary care may help determine appropriate educational material and assess the effectiveness of interventions [6]. Assessment of belief systems and vignettes that depict mental illness have been utilized to tailor public health interventions for the target audience [7], and are associated with increased mental health literacy [8].

### Hispanics in the United States and depression

In the year 2000, first generation Hispanic immigrants accounted for 14.2 million of the Hispanic population (40%) in the United States, second generation U.S. born Hispanics accounted for 9.9 million of the Hispanic population (28%), and third generation U.S. born Hispanics accounted for 11.3% of the population (32%) [9]. By 2020, the overall Hispanic population is expected to grow by 25.7 million, first generation Hispanic immigrants are projected to account for 25% of the Hispanic population growth, second generation U.S. born Hispanics are projected to account for 47% of the Hispanic population growth, and third generation U.S. born Hispanics are projected to account for 28% of the Hispanic population growth [9]. By 2050, Hispanics are projected to make up 24% of the population, compared with 18% of the current population [10, 11].

Depression is a leading cause of disability in the U.S. and Hispanics account for 5.8% of the population with major depression [12, 13]. Data from the National Institute of Mental Health’s Collaborative Psychiatric Epidemiology Surveys (CPES), a representative sample of the population, indicated that U.S.-born Hispanics are more likely to have depression compared to Hispanics born in Latin America and are less likely to follow a treatment plan compared to non-Hispanic whites [14]. Similarly, data from the National Latino and Asian American Study, which included a nationally representative sample of Latinos, indicated that Hispanics who have spent time spent living in the U.S. or were born in the U.S. are more likely to have a psychiatric disorder than non-Hispanic whites [10]. Finally, 1 in 10 Hispanic women living in the U.S. have been diagnosed with a major depressive disorder within the last year [15].

### Stigma and mental health

Concerns about cost of health services can impede access to healthcare, while beliefs about mental health influence a person’s capacity to identify symptoms, and their willingness to seek and to engage in care [5, 13, 16]. Hispanics with lower socioeconomic status and less education are less likely to seek health services, to engage with their medical providers and to discuss behavioral health concerns because of the cultural stigma attached to a mental health condition [16]. Hispanic patients receiving treatment for depression have expressed fear of being stigmatized by their family and deep concerns about depression medication being addictive [17].

### Education level and mental health literacy

HealthyPeople2020, a ten-year set of goals and objectives used as a national guideline to improve health in the U.S., lists education as one of the social determinants of health outcomes [18]. In addition, the amount of education a person has completed has been associated with life span, health literacy, and quality of care received [16, 19, 20]. Lower levels of education have been associated with less use of outpatient care and more psychological distress for men and women, while higher levels of education have been linked to better health, health literacy [21–23] and greater knowledge of mental illness [24]. According to the 2015 Census data on educational attainment, among Hispanic men and women ages 25 or older, 66.7% completed high school or more, 36.8% have some college or more, and 15.5% have a bachelor’s degree or more [25]. While education level or educational attainment are often collected in research, and utilized to understand the demographics of a population or as a control, there is limited research on the role of education level in stigma and depression knowledge [26].

### Integration into healthcare settings

Integrating mental health literacy tools into healthcare settings can improve early detection [27]. By assessing a patient’s health literacy, their attitudes, beliefs and knowledge about a diagnosis, primary care providers can engage patients in early intervention and appropriate mental health services. The combination of age, culturally designed stigma measures, and depression knowledge assessment may lead to more accurate treatment, for Hispanic women in particular [28, 29]. The current study seeks to add to the public health research literature by examining patient-level stigma and knowledge of depression. Specifically, we seek to understand the relationship between stigma, depression knowledge and education level among Hispanic women with depression in primary care.

## Methods

### Setting and participants

This study examines baseline data collected during Project DESEO: Depression Screening and Education: Options to Reduce Barriers to Treatment [28], which took place in a federally qualified health center (FQHC) in Texas over a 24-month period. A total of 350 adult primary care patients were recruited for the larger study [28], and the subsample of adult, Hispanic women were included for analysis in the current study. The clinic provided primary care services to children and adults, including chronic disease management, family planning/women’s health care, and behavioral health care. The geographic area served by the FQHC is comprised of 77% Hispanics [28].

### Procedures

All adult primary care patients were universally screened for depression using the Patient Health Questionnaire-9 (PHQ-9) [30], during annual or new/non-acute visits as part of normal clinical practice. During the enrollment period of February 2015 through October 2016, 18,692 unduplicated patients were seen at the clinic. When a patient scored > 10 points on the PHQ-9, the physician would then initiate a “warm hand off”, wherein the patient was referred and introduced to a Depression Educator and the depression diagnosis confirmed through a clinical interview. During the enrollment timeframe, 1442 patients had a diagnosis of depression and a total of 360 patients met the inclusion criteria for the study - a confirmed diagnosis of depression, self-identified as Hispanic, and not currently in treatment for depression. Ten patients who were offered the opportunity by the Depression Educator, declined to participate in the study. All participants signed an informed consent document prior to participation. The study was reviewed and approved by the Institutional Review Board of the University of Texas at Arlington [28].

### Measures

#### Education level

The education level of participants was collected from the patients’ electronic health record and coded into three categories: (1) less than high school, (2) high school diploma or a General Education Diploma (GED) equivalent, and (3) some college or more, including those who attended trade school, community college, or a university. The electronic health record did not indicate whether education was obtained in the U.S. or in another country.

#### Depression knowledge

The Depression Knowledge Measure (DKM) was developed to assess knowledge of the symptoms and treatment related to depression [31]. The DKM is comprised of 17 questions. The first ten items of the DKM consist of a checklist to identify the five symptoms related to depression from a list of ten possible symptoms, and the last seven items were adapted from a true/false measure for depression literacy to assess depression treatment knowledge [31, 32]. The true or false statements ask if medications can help someone with depression, if depression is a medical condition, if people with depression get better by themselves without professional help, if people with depression should stop taking antidepressants as soon as they feel better, if talking to a counselor can help someone with depression, if antidepressants are addictive, and if antidepressant medications work right away [31]. The DKM is coded for a respondent to receive (0) points for responses answered incorrectly and (1) for responses answered correctly, with total scores ranging from 0 to 17. A higher score is associated with greater knowledge of depression symptoms and treatment.

#### Depression stigma

The Stigma Concerns About Mental Health Care Measure (SCMHC), the Social Distance Measure (Social Distance) and Latino Scale for Antidepressant Stigma Measure (LSAS) are tools used to assess stigma that Hispanics may have about depression [33]. All three measures have been tested in Spanish and English and validated with samples comparable to the population served at the FQHC and recruited for the current study [31, 33, 34].

The SCMHC measure consists of three items designed to measure the role of internalized stigma, fear of stigmatization and stigma from family in seeking mental health treatment [35]. The SCMHC demonstrated acceptable internal consistency within the current sample (α=.75). Responses were coded as (0) disagree, (1) agree, and (7) don’t know/refuse. A respondent received one point for answering (1) agree to a statement. Total scores range from 0 to 3 with higher overall scores indicating an increased internalization of stigma toward mental health care.

The Social Distance measure consists of six items and demonstrated acceptable internal consistency within the current sample (α=.77). The measure assesses the likelihood of the individual to engage with a person who is being treated for depression or has been treated for depression. When Social Distance scores were lowest, an individual was less willing to engage with a person with depression [35]. The Social Distance responses were coded as (0) no, (1) maybe, (2) yes, (7) don’t know/refuse. A respondent received points for every (1) or (2) response, with total scores ranging from 0 to 12. Lower overall scores indicate greater desired social distance from someone who is or has been treated for depression.

The LSAS measure consists of seven items and had good internal consistency within the current sample (α=.84). The measure assessed stigma associated with antidepressant usage and was created specifically for Hispanics using focus groups [35]. The seven questions ask about how they think others may perceive people who take prescription medicine for depression based on things they have heard from family, friends and people around them. The LSAS measure responses were coded as (0) no one thinks that way, (1) some people think that way, (2) everyone thinks that way, (7) don’t know/ refuse. A respondent received points for every (1) or (2) response, with total scores ranging from 0 to 14. Higher overall LSAS scores indicate increased stigma associated with antidepressant usage.

### Data analysis

The data analysis was conducted in SPSS software (v25.0). Bivariate correlations were conducted to assess the association between stigma and depression knowledge. ANCOVA models were used to compare the three stigma measures and depression knowledge by education level while controlling for age of the participant and their depression scores. Correlation analyses used Spearman’ rho (*p*). Assumptions for normality were met for the use of ANCOVA modeling including the normality of predictors in the model and homogeneity of both variance and regression (i.e., no relationship between groups and covariates).

## Results

### Sample

Of the total sample of 350 participants enrolled and consented, complete data for 319 female participants (91.1%) were available on the baseline measures and reported education level. Of the 319 participants, 94.7% were Spanish speaking (*n* = 301). More than half had below a high school education (63.0%, *n* = 201), 22.6% had high school (*n* = 72) and 14.4% had completed at least some college (*n* = 46) (Table 1).

**Table 1:** Characteristics of Participants

Variables	Number (N)	Percentage (%)	M (SD)
Total	319	100.0%	
Age of participant	319		38.41 (10.15)
18 through 24 years old	21	6.6%	
25 through 34 years old	105	32.9%	
35 through 44 years old	116	36.4%	
45 through 54 years old	50	15.7%	
55 through 64 years old	22	6.9%	
65 years old and over	5	1.6%	
Education Level			
Below High School	201	63.0%	
High School	72	22.6%	
Some or more college	46	14.4%	
Spanish Speaking			
Yes	301	94.7%	
No	17	5.3%	
Marital Status			
Married/cohabitating	226	70.8%	
New married	38	11.9%	
Widowed	8	2.5%	
Divorced	24	7.5%	
Other	23	7.5%	
PHQ-9 total	319		38.41 (10.15)
No depression, PHQ-9 score ≦4	0	0.0%	
Mild, PHQ-9 score 5–9	6	1.9%	
Moderate, PHQ-9 score 10–14	58	18.2%	
Moderately severe, PHQ-9 score 15–19	149	46.9%	
Severe, PHQ-9 score ≧ 20	105	33.0%	

### Depression knowledge and stigma

DKM scores were significantly, weakly correlated with the SCMHC (*p* = − .165, *p* = .003), LSAS (*p* = .124, *p* = .028), and Social Distance scores (*p* = .150, *p* = .007). Increased depression knowledge was associated with lower stigma concerns about mental health, greater stigma around psychiatric medication use, and lowered desired social distance for those who are or have been treated for depression (Table 2).

**Table 2:** Correlation between stigma and depression knowledge measures (Spearman’s rho, *p*)

	1. SCMHC	2. LSAS	3. Social Distance
1. SCMHC	–		
2. LSAS	.114*	–	
3. Social Distance	−.148**	.026	–
4. DKM	−.165**	.124*	.150**

* *p* < .05, ** *p* < .01

### Depression knowledge and mental health stigma by education level

Scores on stigma and the DKM measures significantly varied across education level. Education level was divided into three categories: 1) less than high school; 2) high school; and 3) some or more college. Table 3 provides results from each ANCOVA model including age of the participant and their PHQ scores as continuous covariates as well as the outcome scores per educational group. Results from these ANCOVA models indicated individuals with some college education (M = 12.13, SD = 1.92) reported greater depression knowledge (F[2, 312] = 11.82, *p* < .001, partial *η*^2^ = .071) than both the high school (M = 10.86, SD = 1.94) and the less than high school education groups (M = 10.44, SD = 2.16). Similarly, individuals with some college or more education (M = 7.04, SD = 3.22) reported higher mean LSAS stigma of antidepressant usage scores (F[2, 312] = 3.33, *p* = .037, partial *η*^2^ = .015) than the individuals who had less than a high school education (M = 5.74, SD = 3.49), after controlling for age and PHQ-9 scores.

**Table 3:** Differences in depression knowledge and mental health stigma scores by education level, four ANCOVA models (*n* = 319)

				Outcome M (SD)			
Outcome	Predictors in model	F	Partial *η*^2^	Total Sample	Less than HS	HS	Some college or more
SCMHC				0.43 (0.84)	0.48 (0.88)	0.46 (0.86)	0.20 (0.54)
	Education level	2.46^+^	.015				
	Age	4.33*	.014				
	PHQ-9 score	0.98	.003				
LSAS				6.09 (3.46)	5.74 (3.49)	6.47 (3.40)	7.04 (3.22)
	Education level	3.33*	.021				
	Age	0.75	.002				
	PHQ-9 score	2.67	.008				
Social Distance				8.91 (3.12)	8.73 (3.26)	8.72 (2.82)	10.00 (2.77)
	Education level	3.34*	.021				
	Age	0.19	.001				
	PHQ-9 score	0.32	.001				
DKM				10.78 (2.15)	10.44 (2.16)	10.86 (1.94)	12.13 (1.92)
	Education level	11.82**	.071				
	Age	1.69	.005				
	PHQ-9 score	5.94*	.019				

* *p* < .05, ** *p* < .001, ^+^ *p* < .10

The LSAS mean scores for those with a high school education were not significantly different from either group (M = 6.47, SD = 3.40). Mean Social Distance scores significantly differed by education level (F[2, 312] = 3.34, *p* = .037, partial *η*^2^ = .021), after controlling for age and PHQ-9 scores. Individuals who received some college or more education (M = 10.00, SD = 2.77) reported significantly higher mean Social Distance scores than both those with a high school education (M = 8.72, SD = 2.82) and those with less than high school education (M = 8.73, SD = 3.26), after controlling for covariates. Those with some or more college education reported greater willingness to engage with someone who is receiving or had received treatment for depression.

SCMHC score differences between education groups approached significance (F[2, 312] = 2.46, *p* = .087, partial *η*^2^ = .015). In the four models, age was only a significant covariate of SCMHC scores, and PHQ-9 depressions scores significant as a covariate for DKM scores, though each were controlled for across the four models.

## Discussion

In this sample of treatment seeking Hispanic patients at a community-based clinic, we found the more an individual knew about depression and its symptoms, the less likely they were to experience stigma about accessing mental health care services and the more likely they were to engage with a person who has been treated for depression. Unexpectedly, we found higher education was associated with greater stigma about antidepressant use. Typically, low education attainment has been linked with poor quality of life [19, 20] and differences in health outcomes and life expectancy have been almost entirely explained by gaps in education, especially for women [36].

In 2013, an estimated 8.7% of Hispanic adults utilized mental health services and most sought treatment from their primary care provider for depression [13]. Treatment choices vary across ethnic groups; Hispanics are more likely to prefer psychotherapy (46.4%) compared to pharmaceutical therapy (31.6%) and are also more likely to believe that depression medication is addictive than non-Hispanic whites [37, 38]. Limited access to information about depression and about its treatment may lead to somatization of symptoms and poor identification of symptoms [39]. Hispanics with low English proficiency are even less likely to access mental health services, and are more likely to delay care and engagement in treatment [40, 41]. Additional barriers include confidentiality of appointments, taking time to talk to their provider, having an interpreter if they are Spanish-speaking, having a provider who has an understanding of Hispanic culture, and having a provider who has a willingness to learn about their culture and family [42].

Stigma concerns include perceptions of what friends or family might think, and that a person should overcome depression alone [42]. Previous studies of Hispanic patients have found women to be more likely to report having stigma about antidepressant use and therapy, less likely to take medication, and more likely to miss mental health care appointments [43]. Lower stigma about mental health and lower expressed desire to keep social distance from people with depression have been associated with better treatment outcomes, especially among Hispanics [8, 31, 33].

Surprisingly, a significant association between higher education and greater stigma about antidepressant use was found. This unanticipated finding is contrary to previous research which demonstrated a positive relationship between greater education and greater acceptance of medication treatment [44]. Given these findings, greater mental health education may have the unintended consequence of increasing awareness of attitudes that exist in the larger world, thus perpetuating beliefs about the adverse effects of antidepressants. Alternatively, cultural stigma about medication treatment may be so deeply rooted that education level has little influence in reducing the stigma, since all groups reported some stigma associated with antidepressant use [33].

Understood in the context of Jorm’s [5] mental health literacy framework, the public health implications are evident [45]. Labeling of a person with mental illness, related assumptions about safety, and historical mistreatment of people with mental illness have resulted in significant provider and system-level barriers to mental health care [45]. Assessment of recognition, knowledge, and attitudes/beliefs that promote help seeking behaviors can facilitate elimination of patient-level barriers and lead to an increase in the detection of mental illness and early engagement into treatment [3]. A person’s willingness to accept treatment for depression is largely influenced by contextual attitudes associated with having depression and what their support network, such as family and friends, may think or know about depression [46].

Findings lend support to the construct that disease literacy is an essential component to treatment engagement. Early assessment of the education level of patients could be beneficial at the point of care as it may help provide insight into intervention options and reduce barriers associated with mental health treatment. Along with a screening for depression, stigma and knowledge measures collected during initial visits, or prior to deciding on treatment, can benefit the patient, as well as the provider. Tools that are culturally effective, gauge mental health literacy, and engage patients through education can offer clinical guidance on discussing diagnoses with a patient and may be necessary for proper treatment adherence [24].

### Limitations

The results of this study should be interpreted in light of several limitations. First, the data for education level lacked granularity below the high school level. Given that the majority of participants (63.7%) had less than a high school education, more precise data for education level could have improved understanding of the findings. Additionally, since the participants were primarily Spanish-speaking, we were unable to examine differences in mental health literacy and stigma between Spanish- and English-speaking participants. Language proficiency, as well as immigration status and number of years in the United States, are often indicators of acculturation, and have been associated with increased stigma towards mental health treatment [47, 48]. This sample also consisted of Hispanic women and is, therefore, limited in its generalizability to Hispanic men whose mental health help-seeking attitudes are influenced by masculine norms and self-stigma [49].

Due to funder requirements, a control group was not utilized in this study, which introduces the possibility of selection bias. All participants who were enrolled in this study were experiencing depression and willing to meet with the Depression Educator regarding their depression diagnosis. And, while it is well-established that collecting measures via self-report leads to more accurate assessment of depressive symptoms [30], the possibility of reporting bias exists. Finally, studies using a more heterogenous Hispanic sample, including the use of a non-depressed Hispanic control group, should be conducted in order to examine important differences in mental health literacy and stigma that may exist between these groups.

## Conclusions

Stigma continues to be difficult to eradicate or prevent, despite education. Stigma and knowledge screening measures are indicators that can guide best practices and help evaluate effective mental health literacy initiatives [50]. Findings from this study support previous research which suggests mental health literacy can mitigate stigma’s adverse impact on care seeking [51], and that mental health literacy varies by education level, with higher education resulting in higher mental health literacy.

Strategies for improving mental health literacy in health care settings include assessment of education level, culturally relevant care, and family engagement in treatment decisions [51]. For purposes of better practice, healthcare providers should understand the role of stigma in engagement in treatment. A culturally effective, patient-centered environment in which people can comfortably share mental health concerns, and in which patient education addresses myths about depression and its treatment, is essential [43].

Culturally adapted educational materials have demonstrated success in changing recognition, knowledge, and beliefs through empathy and association [31]. Early intervention in the education system through research and policy holds promise for improving mental health literacy, preventing or managing early onset of disease, and engaging youth in elimination of stigma [52] Future research of a qualitative nature could provide a deeper understanding of the Hispanic community’s knowledge of depression symptoms, treatment and belief systems that influence engagement into care, social engagement with someone who has depression, and antidepressant use.

## Abbreviations

ANCOVA: Analysis of covariance
CMS: Center for medicare and medicaid services
DESEO: Depression screening and education: options to reduce barriers to treatment
DKM: Depression knowledge measure
FQHC: Federally qualified health center
GED: General education diploma
LSAS: Latino scale for antidepressant stigma
M: Mean value
PHQ-9: 9-item patient health questionnaire
PI: Principal investigator
SCMHC: Stigma concerns about mental health care
SD: Standard deviation value
